# Drawbacks for Studies on the Essential Oil of *Dracocephalum kotschyi*

**DOI:** 10.21315/mjms2023.30.1.18

**Published:** 2023-02-28

**Authors:** Le Pham Tan Quoc

**Affiliations:** Institute of Biotechnology and Food Technology, Industrial University of Ho Chi Minh City, Ho Chi Minh City, Vietnam

Dear Editor,

Based on the study by Zarei-Yazdeli et al. (1), the methanolic extract of *Dracocephalum kotschyi* (*D. kotschyi*) possesses antibacterial activity and also has a better synergistic antibacterial effect when combining with extract of other plants, particularly *Trachyspermum ammi*. Many have raised questions about the bioactivity of the phytochemical compounds of *D. kotschyi* and the combination or interaction between these compounds and other agents to enhance their bioactivity.

Nowadays, phytochemical compounds of *D. kotschyi* have been shown to exist in both essential oil (EO) and extract isolated using different solvents (methanol, distilled water, etc.) (1, 2). Previous studies have pointed out that extract of *D. kotschyi* possesses numerous bioactive compounds, such as flavonoids, phytosterols, saponins, tannins and phycobatannin. This material was generally used to biosynthesise nanoparticles (NPs) (e.g. Drac-AgNPs and Drac-AuNPs) (2, 3), which have many benefits: owing to their reduced toxicity they are beneficial for human health and biomedical applications (3); furthermore, their antibacterial, cytotoxicity, haemocompatibility and catalytic properties are significantly improved (2).

In fact, except for extract, the chemical components of EO of *D. kotschyi* have also been carefully studied. Ghavam et al. (4) found 21–28 active compounds in the EO of *D. kotschyi*, depending on the source of the initial sample (cultivated or wild plant). Compared to other materials, the yield of EO extraction was quite low (0.2%–0.97%). The main chemical components in the EO were monoterpenes hydrocarbons, oxygenated monoterpenes, sesquiterpene hydrocarbons, oxygenated sesquiterpenes and others. However, so far, the bioactivity of the EO has been little studied, apart from the antibacterial activity (4) and antinociceptive effects in mice (5), whereas the bioactivity of the extract has been widely studied, showing antioxidant, antibacterial, anticancerous, antinociceptive, antihyperlipidaemic, antispasmodic, cytotoxic and immunomodulatory effects (6). This shows that the application of *D. kotschyi* EO in the medical field has many restrictions.

In addition, compared to the extract, one of the most exciting things about *D. kotschyi* EO is that there is no official standard and also no published reports on the biosynthesis of NPs using *D. kotschyi* EO as the reducing agent. In my opinion, these are important gaps that future studies should focus on filling. Furthermore, in most previous studies, the hydrodistilled EOs are obtained from aerial parts of *D. kotschyi*, but EOs can also exist in the leaves, branches or flowers. However, the separation process for these parts may be difficult and require many cycles. Therefore, there are many important questions to be answered in the future, such as: Which parts have the highest EO content? What are the differences in the chemical components for the different parts? What are the bioactivities of these chemical components? Can we synthesise NPs from EOs?

To date, numerous reports on *D. kotschyi* EO are available but there are still many interesting things to discover, particularly the bioactivity of EO for human health and the potential biosynthesis of NPs. Based on the opinion mentioned above, I strongly believe that the obtained results can enhance our knowledge and would be applied widely in the pharmaceutical and medical fields.
